# Investigation of the protective and therapeutic effects of *Lactobacillus casei* and *Saccharomyces cerevisiae* in a breast cancer mouse model

**DOI:** 10.3934/microbiol.2022016

**Published:** 2022-05-16

**Authors:** Kholoud Baraka, Rania Abozahra, Maged Wasfy Helmy, Nada Salah El Dine El Meniawy, Sarah M Abdelhamid

**Affiliations:** 1 Microbiology and Immunology Department, Faculty of Pharmacy, Damanhour University, Damanhur, Egypt; 2 Pharmacology and Toxicology Department, Faculty of Pharmacy, Damanhour University, Damanhur, Egypt

**Keywords:** *Lactobacillus casei*, *Saccharomyces cerevisiae*, mouse, breast cancer, cytokines

## Abstract

**Introduction:**

The development of novel strategies for cancer therapy is crucial to improve standard treatment protocols.

**Aim:**

This study aimed to determine the protective and therapeutic effects of heat-killed preparations of *Lactobacillus casei* and *Saccharomyces cerevisiae* in a breast cancer mouse model.

**Methods:**

Forty-two female BALB/c mice (7–8 weeks old) were divided into six groups (seven mice per group). Four groups were injected with 10^7^ Ehrlich ascites tumor (EAT) cells suspended in phosphate-buffered saline (PBS) subcutaneously into the left side of the mammary fat pad. Tumor growth was monitored weekly until all animals developed a palpable tumor. The tumor-bearing mice in the experimental groups received heat-killed *L. casei* or *S. cerevisiae* three times per week for 35 days. The mice in the control group received PBS. The remaining two groups received heated *L. casei* or *S. cerevisiae* and then were injected with EAT cells. After 35 days, all mice were sacrificed to determine the immune response.

**Results:**

Animals that received heated *S. cerevisiae* exhibited the lowest rate of tumor growth compared with the other groups. TGF-β and IL-4 secretion was increased in all mice, whereas the secretion of INF-γ and IL-10 was decreased in breast tissues. Moreover, at the histopathological level, the volume of viable tumor in the control group was higher than in the treated groups.

**Conclusion:**

Supplementary treatment with *S. cerevisiae* resulted in the best outcome in the breast cancer model compared with other treated and vaccinated groups.

## 1. Introduction

Cancer significantly is a major cause of death affecting the overall life expectancy worldwide [1]. According to the World Health Organization (WHO) estimates in 2019, cancer is the second leading cause of death for Egyptians younger than 70 years [1],[2]. In 2020, the Global Cancer Observatory reported an incidence of 2.26 million breast cancer cases and 684,996 (6.9%) deaths [3]. Breast cancer treatment protocols are variable as they depend upon several factors including: the histological diagnosis of the patient, age, disease stage, and previous therapeutic regimens. Breast cancer treatment includes surgery, radiotherapy, and chemotherapy. Several anticancer drugs have been approved for the treatment of breast cancer; however, poor permeability and aqueous solubility of those drugs may occur. Therefore, the targeted delivery and controlled release of anti-cancer drugs may be achieved using nanoparticles, which enhance therapeutic efficacy and reduce adverse effects. Immunotherapy is another effective approach for cancer treatment. It acts by boosting the body's natural defences to destroy cancer cells [4]. The tumor micro-environment consists of various inflammatory cells including cancer-associated fibroblasts, stromal cells, tumor-associated macrophages, infiltrating immune cells, pericytes, and endothelial cells, which comprise the tumor vasculature. These inflammatory cells participate in the cancer-related inflammation as well. Tumor cells communicate with inflammatory cells by secreting cytokines and growth factors to stimulate both tumor growth and resistance to chemotherapy [5].

Interferons are a group of pleiotropic cytokines that have multiple roles in cells' communication during the innate and acquired immune response, host defense against viral and bacterial infections, and tumor surveillance [6]. Interferon-gamma (IFN-γ) either was reported to stimulate or suppress the natural killer cell-mediated lysis of tumor cells derived from various pediatric tumor cell lines [7]. Treatment of tumor cell lines with IFN-γ induced differential upregulation of major histocompatibility complex (MHC) class I, which determines tumor cell sensitivity or resistance to natural killer (NK) cell-mediated lysis [6]. Additionally, interleukin (IL)-4 and IL-10 are pleiotropic cytokines that are important components of the humoral immune response. They play an immunosuppressive role in the cellular immune response. Moreover, it was reported that IL-4 and IL-10 have a direct anti-proliferative effect on some tumor cells including breast carcinomas,which may be because of their ability to increase the host antitumor response [8]. IL-10 is secreted by different cell types including macrophages and T cells [8],[9]. Several studies reported that IL-10 was associated with the progression and promotion of tumors, whereas other studies have indicated its role in the elimination and suppression of angiogenesis and metastasis. IL-10 is believed to enhance tumor immune escape by reducing the antitumor immune response in the microenvironment, which is indicated by the positive correlation between IL-10 levels and the poor prognosis of patients [9],[10]. Tumor growth factor-β (TGF-β) is a member of a large family of structurally related cytokines that includes bone morphogenetic proteins, growth and differentiation factors, activins, and inhibins. It has an important role in numerous cellular processes including proliferation, apoptosis, adhesion, differentiation, motility, and immune regulation [10],[11]. The “TGF-β paradox” describes a switch in the role of TGF-β from being tumor suppressive during early-stage tumors to tumor promoting in later-stage tumors [12].

It is necessary to search for agents that induce apoptosis of cancer cells with minimal side effects. One of these agents is the *Lactobacillus casei* (*L. casei*) probiotic strain, which is an ingredient in food supplements in many countries. The administration of this probiotic fermented milk was reported to be associated with the decrease in tumor angiogenesis, tumor size, and extravasation of tumor cells [9],[13]. The daily intake of *L. casei* was reported to improve the adaptive immune response and the cytotoxicity of natural killer cells in mice bearing invasive ductal carcinoma [14],[15]. A second agent is the *Saccharomyces cerevisiae* (*S. cerevisiae*); the baker's yeast. It was reported that an intra-tumoral injection with heat-killed *S. cerevisiae*, induced significant levels of apoptosis in breast cancer-bearing mice. Yeast cells were reported to exert anticancer activity by two independent mechanisms: 1) an apoptotic effect and 2) immunomodulatory effects. Additionally, significant tumor regression was reported following the injection of *S. cerevisiae* into mice. Moreover, yeast-treated tumor-bearing mice exhibited a thinner layer of viable tumor tissue compared with the control group [16].

## 2. Materials and methods

### 2.1. Animals

Forty-two female BALB/C mice (7–8 weeks, approximately 10 g) were maintained in pathogen-free stainless steel mesh cages. They were divided into six groups of seven mice per group with one group per cage. A balance was used to weigh each mouse. They were provided with autoclaved and non-fluorescent mouse chow and water under standard conditions of light, relative humidity, and temperature [16],[17].

### 2.2. Preparation of *Saccharomyces cerevisiae*

Commercially available baker's and brewer's yeast (Saf-instant, Egypt) containing *S. cerevisiae* were solubilized in phosphate-buffered saline (PBS) (Biowest, France), transferred to Sabaroud's Dextrose agar (Merck Millipore, USA), and cultured at 37 °C for 24 h. The off-white colonies were collected and washed with PBS. Suspensions of *S. cerevisiae* were heat-killed by incubation at 90 °C for 1 h and washed three times with PBS. Quantitation was conducted using a hemocytometer (JSQA, China). Cell suspensions were adjusted to 1 × 10^9^ and 1 × 10^6^ cells/mL [18],[19].

### 2.3. Preparation of *Lactobacillus casei*

*Lactobacillus casei* (ATCC 393, EMCC number 1093 T) was purchased from Cairo Mircen, Egypt. Bacteria were cultured anaerobically with 5% CO_2_ for 48 h. The pH was adjusted to 6.2–6.5 on a de Man, Rogosa, and Sharpe agar (Merck Millipore, USA). Bacterial colonies were washed with PBS and were heat-killed at 56 °C for 60 min. Quantitation was conducted using a hemocytometer. Cell suspensions were adjusted to 1 × 10^6^ cells/mL [15].

### 2.4. Development of the tumor model

Ehrlich ascites tumor (EAT) cells were purchased from the National Institute of Cancer, Egypt. Tumor cells were collected from the ascites fluid of Balb/c mice harbouring 8–10 day-old tumors. Approximately 10^7^ EAT cells were suspended in 0.2 mL PBS and were injected subcutaneously into the left side of the mammary fat pad of BALB/c female mice of five groups. Tumor growth was monitored weekly using digital calipers. Tumor volume was determined by measuring both perpendicular diameters of the tumor using a micrometer according to the following equation: Tumor volume = (4/3) π (minor axis)^2^ × major axis [20]–[22].

### 2.5. Experimental design and tumor transplantation

To investigate the *in vivo* antitumor efficacy of the heat-killed *L. casei* and *S. cerevisiae*, 42 tumor-bearing mice were divided into six groups (seven mice/group): Group I: the control group consisting of breast cancer-bearing mice injected intra-tumorally with 100 µL of PBS three times per week; Group II: breast cancer-bearing mice injected intra-tumorally with 100 µL of 10^6^ cells/mL of *S. cerevisiae* three times per week; Group III: breast cancer-bearing mice injected intra-tumorally with 100 µL of 10^9^ cells/mL of *S. cerevisiae* three times per week; Group IV: breast cancer-bearing mice injected intra-tumorally with 100 µL of 10^6^ cells/mL of *L. casei* three times per week; Group V: mice injected subcutaneously with 100 µL of 10^6^ cells/mL of *S. cerevisiae* before EAT injection three times per week; and Group VI: mice injected subcutaneously with 100 µL of 10^6^ cells/mL of *L. casei* before EAT injection three times per week.

Tumor growth was monitored and assessed weekly during treatment days as previously described. All treatment protocols were initiated on day 5 after tumor induction, whereas all immunization protocols began immediately after purchasing mice and before tumor induction. After 35 days of developing a palpable tumor, the mice were sacrificed. Blood samples were collected from the retro-orbital plexus of the mice under anesthesia using thiopental. Plasma samples were analyzed for cytokines immediately. The mice were then euthanized with a high dose of thiopental (50 mg/kg). Tumor tissues were removed, immediately chilled on ice, and weighed. Tumors were then divided into two parts: one part was weighed and stored at −80 °C until the day of assessment, whereas the other portion was subjected to histopathological evaluation and immune-histochemical analysis of Ki67 protein [15]–[17].

### 2.6. Cytokine analysis

#### 2.6.1. Plasma sample collection

Plasma samples were collected on day 35 following tumor cell inoculation from all mouse groups through the retro-orbital sinus for determination of TGF-β, IL-4, IL-10, and IFN-γ levels using a mouse ELISA kit (Nova, Beijing, China).

#### 2.6.2. Detection of tumor growth biomarkers in tissue homogenates

Tumors were homogenized in cold PBS to obtain a final 40% tissue homogenate, which was divided into four aliquots for the measurement of TGF-β, IL-4, IL-10, and IFN-γ levels using an Enzyme linked immune-sorbent assay (ELISA).

#### 2.6.3. Histo-pathological study and average necrosis scoring

Tumor samples were preserved and fixed in 10% neutral formalin for 24 h at room temperature. Five-µm-thick sections were placed in distilled water, stained with hematoxylin for 5 min and eosin for 2 min, dehydrated with alcohol, mounted in Canada balsam, and examined microscopically. Necrosis of the excised mammary tumor was assessed semi-quantitatively by examining 10 random sections (×40) from each excised tumor and scoring was done on a scale from 1 to 4 for the following criteria: Score 4, section of poorly differentiated tumor showing >50% necrosis; Score 3, section of poorly differentiated tumor showing nearly 35% necrosis; Score 2, section of poorly differentiated tumor showing nearly 25% necrosis; and Score 1, section of poorly differentiated tumor showing nearly 10% necrosis. The mean value of all 10 scores was determined for each excised tumor and expressed as the mean of the necrosis scale ± SE [20],[23],[24].

#### 2.6.4. Immuno-histochemical assessment of KI-67 protein in mammary tumor

Mammary tumor sections were further immunohistochemically stained using an avidin-biotin-peroxidase complex [20]. Briefly, after inhibiting endogenous peroxidases by 3% hydrogen peroxide, slides were treated with microwaves in 10 mM citrate buffer for 15 min. Slides were then washed by Tris-buffered saline (TBS) and were incubated with 10% normal goat serum at 23 °C for 30 min. Slides were then incubated at 4 °C overnight with 200 µg/mL prediluted mouse KI-67 monoclonal antibodies (Cat#RM-9106-R7, Thermo Fisher Scientific Fremont, USA). In the next day, biotin-conjugated goat anti-mouse or anti-rabbit IgG (diluted 1:200) were added and left for 20 min. A final incubation with streptavidin conjugated HRP horseradish peroxidase (diluted 1:200 in TBS) was conducted for 45 min. The reaction product was developed by adding diaminobenzidine for 5 min.

#### 2.6.5. Evaluation of KI-67

In order to measure the protein expression, immuno-histochemical signals of the proliferating cell nuclear antigen and Ki-67 were quantified using a digital imaging technique. The Image J software (version 1.45s) program and computer-assisted microscopy were used. This technique involved the transformation of the captured colored images into gray scale by changing the image type from colored into an 8-bit type. The image quality was enhanced using the median filter. The area of the immuno-histochemical reaction was selected using oval selection tools, thresholding the binary process, and then foreground pixels were converted into black colour and the background pixels into white colour. The resulting image was then analysed by obtaining the histogram of the numbers of pixels of black and white areas. The degree of positivity was estimated by the percentage of black pixels in the binary image compared with the total number of pixels in the selected area. Protein expression was estimated from the average of 10 random sections (×100) from the mammary tissue of each treated animal [21],[23],[24].

#### 2.6.6. Statistical analysis

Statistical analysis of the *in vivo* antitumor activity was conducted using IBM SPSS software package version 20. For pair-wise comparisons between the studied groups, analysis of variance test and Tukey's multiple comparisons test were conducted. The significance of the results was designated at the 5% level.

**Figure 1.**
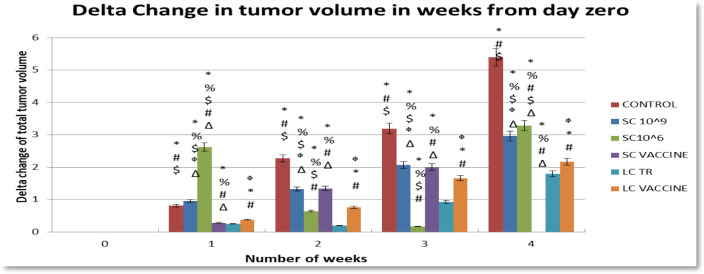
The change of tumor growth in the six mice groups.

## 3. Results

**Figure 2.**
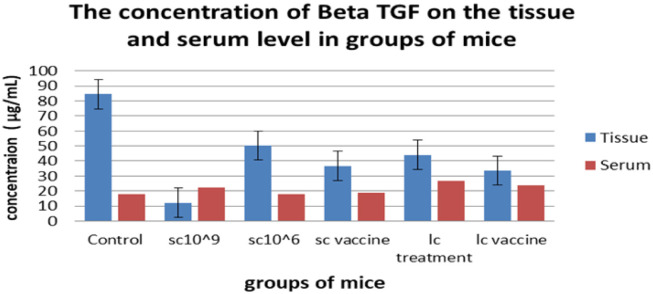
The serum and tissue levels of TGF-β of all treated groups.

**Figure 3.**
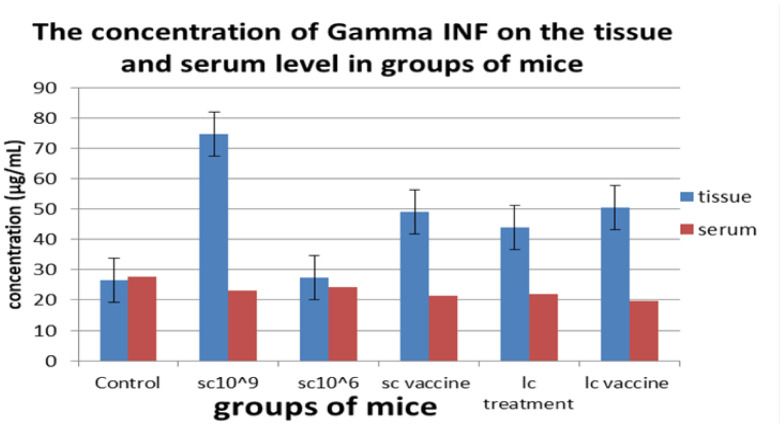
The serum and tissue levels of INF-γ of all treated groups.

**Figure 4.**
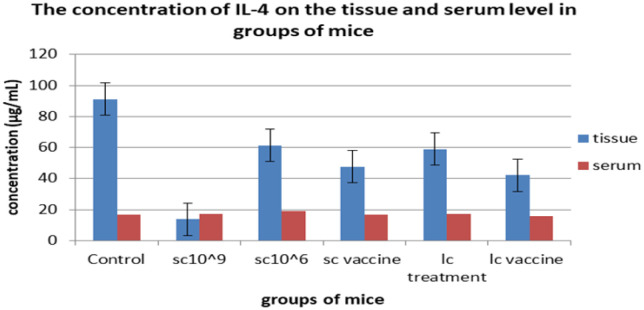
The serum and tissue levels of IL-4 of all treated groups.

**Figure 5.**
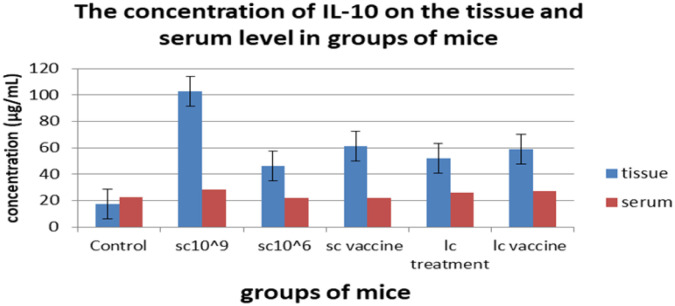
The serum and tissue levels of IL-10 of all treated groups.

Tumor growth was calculated as the delta change, which is the change in tumor volume throughout the experiment versus the beginning. The mice receiving 100 µL of 10^6^ cells/mL of *L. casei* exhibited the smallest delta change in tumor growth among all groups. On the other hand, the tumor growth in the control group and the tumor-bearing mice receiving *S. cerevisiae* (10^9^ cells/mL) groups was higher than the other groups; however, tumor-bearing mice receiving *S. cerevisiae* groups (10^6^ cells/mL) showed regression in tumor growth in weeks 2 and 3, although this effect failed to be maintained in week 4 (Figure 1). The effects of yeast and *Lactobacillus* for either treatment or vaccination (3 times/week) on the tissue and serum levels of TGF-β, IL-4, IL-10, and IFN-γ were measured on day 35 of tumor cell transplantation (Figures 2–5). Cytokines were assayed in all groups. Regarding TGF-β, a minor change was observed in serum levels in the *L. casei*-treated mice; however, it was almost the same in the other groups (Figure 2). At the tissue level, however, a marked decrease in TGF-β levels was observed compared with the control group. The lowest TGF-β tissue levels were observed in the tumor-bearing mice receiving *S. cerevisiae* (10^6^ cells/mL). Regarding IFN-γ, an undetectable change was observed in the serum levels in all groups. In contrast, a marked increase in IFN-γ tissue levels was observed in all groups. The maximum level was observed in the tumor-bearing mice receiving *S. cerevisiae* (10^9^ cells/mL), and the minimum level was observed in the control group (Figure 3). Regarding IL-4, minor changes were observed in the serum levels in mice treated with *S. cerevisiae* (10^6^ cells/mL) compared with the other groups. Conversely, a remarkable decrease of IL-4 tissue levels was observed in all groups compared with the control group. The lowest tissue levels of IL-4 were observed in the tumor-bearing mice receiving *S. cerevisiae* (10^9^ cells/mL) (Figure 4). Moreover, a minor change was observed in IL-10 serum levels in *S. cerevisiae* (10^6^ cells/mL)-treated mice, whereas it was almost the same in other groups. In contrast, a marked increase was observed in the tissues. The highest increase was observed in the tumor-bearing mice receiving *S. cerevisiae* (10^9^ cells/mL), and the lowest level was observed in the control group (Figure 5).

### 3.1. Histopathology

**Figure 6.**
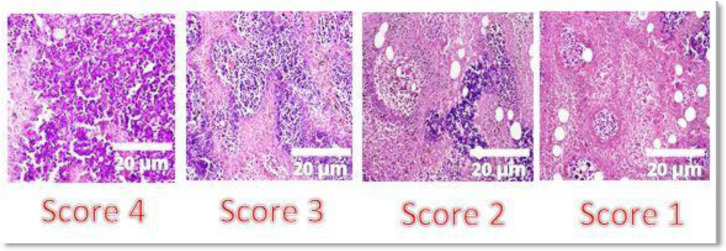
Histopathological sections for all groups. *Note: Score 1: poorly differentiated tumor showing approximately 10% necrosis; score 2: poorly differentiated tumor showing 25% necrosis; score 3: poorly differentiated tumor showing 35% necrosis; and score 4: poorly differentiated tumor showing >50% necrosis.

**Figure 7.**
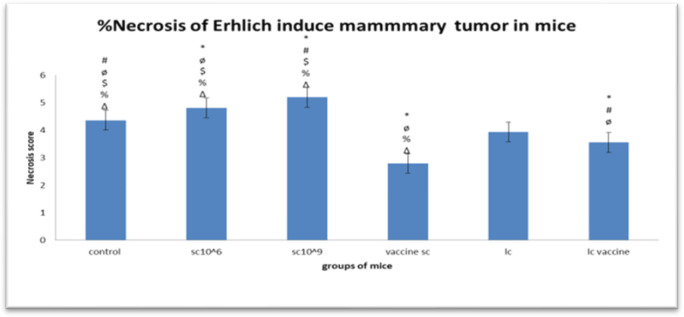
Necrosis scores for mice groups. *Note: p < 0.05 vs. Positive Control, # p < 0.05 vs. SC106, ø p < 0.05 vs. SC109, $ p < 0.05 vs. Vaccine SC, % p < 0.05 vs. IC, Δ p < 0.05 vs. IC vaccine.

Tumor sections from all groups were prepared for histopathological examination. Histopathology of the tumor sections from *S. cerevisiae*- and *L. casei*-treated and vaccinated tumors revealed tumor regression. Besides, extensive degenerative changes in the tumor tissue, such as karyolysis (fading of chromatin) and karyorrhexis (fragmentation of nuclei) were observed. Tumor sections from all groups showed poorly differentiated carcinoma infiltrating into the skeletal muscles and adipose tissue. Viable tumor was also observed at the periphery and ischemic tumor was observed at the center. The volume of viable tumor in the PBS-treated group was greater than that of the other groups (Figure 6). It was found that the control group corresponded to a score of 4; the *Saccharomyces* vaccine corresponded to a score of 3; *Lactobacillus* treatment, *Lactobacillus* vaccine, and *Saccharomyces* (10^9^ cells/mL) corresponded to a score of 2; and *Saccharomyces* (10^6^ cells/mL) corresponded to a score of 1 (Figure 7).

### 3.2. Ischemic coagulative necrosis and degenerative changes

The H&E-stained sections for the mice groups exhibited ischemic (coagulative) necrosis in the center of the tumors; however, there were more residual islands of viable tumor in the PBS-treated group than in the other groups. Compared with the control group, all treated groups showed lower average tumor necrosis scores, but to a different extent, whereas the *S. cerevisiae* (10^6^ cells/mL) treated group had the lowest average necrosis, which was equal to 2.17.

**Figure 8.**
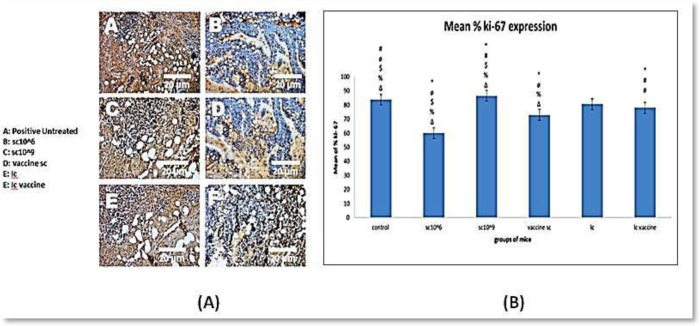
(A) Ki-67 levels in different groups, (B) the mean % Ki-67 expression. *Note: Where * p < 0.05 vs. Positive Control, # p < 0.05 vs. SC10^6^, ø p < 0.05 vs. SC10^9^, $ p < 0.05 vs. Vaccine SC, % p < 0.05 vs. IC, Δ p < 0.05 vs. IC vaccine.

Ki 67 was evaluated in the different mouse groups (Figure 8). From all previous data, *S. cerevisiae* (10^6^ cells/mL) exhibited the fewest proliferation islands, whereas both the control group and the group receiving *S. cerevisiae* (10^9^ cells/mL) showed the highest level of cell proliferation. However, the other groups exhibited a significant difference from the control and the group that received *S. cerevisiae* (10^9^ cells/mL).

## 4. Discussion

Treatment for breast cancer, the second leading cause of death in females worldwide, is complex and variable depending on several factors. Standard treatment includes surgery, radiation therapy, chemotherapy, hormonal therapy, targeted drugs, and immunotherapy [4]. In the present study, we determined the protective and therapeutic effects of heat-killed preparations of *L. casei*, a probiotic found in food and fermented milk, and *S. Cerevisiae*, a type of yeast used for baking against breast cancer. We established an Ehrlich cell model of breast cancer *in vivo*, examined the tumor histologically, and measured biomarkers after 35 days. We tested different concentrations of *S. cerevisiae* and *L. casei* on breast cancer-bearing mice, and their anti-cancer effects as a treatment or vaccine were determined. Results indicated that an intra-tumoral injection of heat-killed *L. casei* caused significant tumor regression compared with the other groups. In addition, we used a heat-killed preparation of *L. casei* to induce signals for the induction of a robust cellular immunity against tumor antigens. Previous studies have suggested that *L. casei* affects the immune response by upregulating surface MHC class II and CD86 on dendritic cells (costimulatory molecules) and promotes IL-12 secretion (a potent polarizing cytokine of the Th1 immune response) [25]–[27]. In this study, results showed that after receiving the *S. cerevisiae* vaccine, tumor regression was observed compared with the control group. This might occur because both dendritic cells and macrophages are known to take up *Saccharomyces* for processing and presentation to the immune system, which resulted in cell and cytokine-mediated effects [17]. Recent studies have shown that beta-glucan, derived from the cell wall of *S. cerevisiae*, exhibits potent NK enhancement effects [28]. Natural killer cells represent a major component of innate immunity that plays an important role in immune surveillance against cancer [16],[17],[29].

Tumors can evade immune responses by secreting cytokines, such as IL-4, TGF-β, IL-10, and IFN-γ. These cytokines can suppress the antitumor immunity including inflammatory macrophages and the Th1 response [25],[26]. The “TGF-β paradox” explains how TGF-β switches from a tumor suppressor at early stages of tumor growth to a tumor promoter at later stages [12]. As tumor progression proceeds, the cells often lose the capability of responding to TGF-β-mediated growth inhibition. TGF-β signalling may be used instead to increase the epithelial–mesenchymal transition, invasion, and metastasis [14]. This explains the elevation of TGF-β levels in the control group tissues compared with other groups at the scarifying step in the current study. However, at the serum level, the control group showed somewhat lower levels. In tissues, mice administered *S. cerevisiae* (10^9^ cells/mL) exhibited downregulated TGF-β levels compared with the other groups, especially the control group, which initially had the highest levels [22].

IFN-γ levels are associated with the antitumor response [28]. Consistent with our data, this study showed that IFN-γ was markedly increased in the tumor-bearing mice receiving the heat-killed *S. cerevisiae* (10^9^ cells/mL) compared with the control group on tissue levels. In contrast, another study reported that IFN-γ levels did not show any significant changes between control group mice and mice administered heat-killed *L. casei* [15]. However, based on the IFN-γ serum levels, the effect was dose-dependent and the IFN-γ levels were not significantly different among the groups. This might be due to the high infection dose that led to cytokine consumption [16],[17],[22].

IL-4 is a pleiotropic cytokine that has two important roles: it promotes the proliferation of immunoglobulins and their class switching [8], and it inhibits the growth of cancer cells *in vitro*, which may be due to its ability to increase the host antitumor response. This occurs primarily through the upregulation of MHC class I and II antigens by IL-4 [30],[31] which could explain its higher serum levels in both treated and vaccinated groups than the control group. In contrast, IL-4 tissue levels were higher in the control group compared with the treated and vaccinated groups because of a decline in the proliferation of cancer cells, which is consistent with the results reported by Jafari *et al*. [17].

With respect to IL-10, our results indicated that its tissue levels were elevated in treated and vaccinated groups compared with the control group. This may result from its ability to enhance tumor immune escape by reducing the antitumor immune response in the microenvironment [22]. This occurs by reducing or inhibiting antigen presentation through down-regulation of both MHC class II expression [32] in APCs as well as MHC class I in tumor cells [33]. In contrast, a minor change was observed in its serum levels compared with the control group and other groups. Other studies demonstrated a significant decrease in IL-10 levels in both *L. casei* and *S. cerevisiae*-treated mice [16],[17].

Histopathology of both *S. cerevisiae*- and *L. casei*-treated and vaccinated tumors showed tumor regression that was further coupled with extensive degenerative changes in the tumor tissue including karyolysis (fading of chromatin) and karyorrhexis (fragmentation of nuclei). In addition, histopathology showed ischemic necrosis of the central part, leaving thin layers of tumor cells at the center and periphery. This resulted from inadequate or no blood supply from the excessive growth of the tumor cells. The control tumor-bearing mice exhibited many sheets of viable tumor cells scattered between necrotic tissues. These changes were associated with a tumor growth curve demonstrating a significant antitumor response and peaked at 35 days, which is consistent with the results reported by Ghoneim *et al*. [18].

By calculating the percentage of tumor necrosis among the groups, we observed that all treated groups demonstrated significantly lower average tumor necrosis scores compared with the control group, but to a different extent, whereas the *S. cerevisiae* (10^6^ cells/mL)-treated group showed the lowest average necrosis (2.17). In contrast, other studies reported that the necrosis level was elevated in groups treated with *Saccharomyces*, especially those receiving higher doses [16]. By evaluating the results of Ki67 expression, which is indicative of cell proliferation, we found that the group treated with *S. cerevisiae* (10^6^ cells/mL) had the fewest proliferation islands, whereas both the control group and the group receiving *S. cerevisiae* (10^9^ cells/mL) exhibited the highest levels of cell proliferation. In contrast, another study revealed that the administration of probiotics reduced Ki67 levels compared with the control group [34],[35].

Up to our knowledge, this is the first Egyptian study evaluating the protective or curative effects of *L. casei* in a murine breast cancer model. Supplementary treatment with *S. cerevisiae* (10^6^ cells/mL) revealed the best histopathological outcome compared with the other treated and vaccinated groups. However, at the immunological level, *S. cerevisiae* (10^6^ cells/mL) showed better results compared with the control group. Moreover, *L. casei* demonstrated better results than the control group indicating that administration of complementary therapy of yeast or probiotics can inhibit or slow the tumor progression and enhance the immune response.
